# Chloroform Narcosis

**Published:** 1894-12

**Authors:** 


### Chloroform Narcosis.

Resuscitation in chloroform narcosis has been accomplished by a new method devised by Maas, one of Kœnig's assistants, of Gottingen. In the first case the ordinary means of resuscitation had been tried for an hour without effect; respiration and pulse had entirely ceased. Maas then made rapid rhythmical compressions—120 per minute—of the cardiac region, whereupon the heart's action gradually increased and the patient recovered. A second severe case responded with the same result to the treatment. Maas ascribes the effect of the cardiac compressions to the driving of the blood into the larger arteries.—*Medical and Surgical Reporter.*

